# Fc gamma receptor binding modulates IgG clearance in cancer cachexia

**DOI:** 10.3389/fimmu.2026.1676732

**Published:** 2026-03-31

**Authors:** Bryan C. Remaily, Kyeongmin Kim, Justin Thomas, Adeoluwa Adeluola, Greg Young, Min Hai, Mayu Fukuda, Gillian Mulcahy, Camille Stanton, Lauren Granchie, Pankaj Kumar, Trang Vu, Faith Jeffrey, Noah Palozzi, Xiaokui Mo, Samuel K. Kulp, Dwight H. Owen, Thomas A. Mace, Christopher C. Coss, Mitch A. Phelps, Latha P. Ganesan

**Affiliations:** 1 Division of Pharmaceutics and Pharmacology, College of Pharmacy, The Ohio State University, Columbus, OH, United States; 2 Department of Internal Medicine, The Ohio State University, Columbus, OH, United States; 3 Department of Biomedical Informatics, The Ohio State University, Columbus, OH, United States; 4 Division of Medical Oncology, The Ohio State University James Comprehensive Cancer Center, Columbus, OH, United States; 5 The Ohio State University – James Comprehensive Cancer Center, Columbus, OH, United States; 6 The Pelotonia Institute for Immuno-Oncology, The Ohio State University, Columbus, OH, United States; 7 Division of Gastroenterology, Hepatology & Nutrition, Department of Medicine, The Ohio State University, Columbus, OH, United States

**Keywords:** antibody pharmacology, cancer cachexia, catabolic clearance, Fc receptors, FcγR, immune checkpoint inhibitors

## Abstract

**Background:**

Patients with cancer-cachexia display a general resistance to Immune Checkpoint Inhibitor (ICI) therapy, as well as an elevated baseline catabolic clearance (CL) of ICIs, which serves as a prognostic indicator of overall survival independent of dose and drug exposure. Increased rate of ICI CL is present in the Lewis Lung Carcinoma (LLC) murine model of cachexia, but absent in the non-cachectic MC38 model. Fc-Gamma Receptors (FcγRs) bind the Fc portion of antibodies and can impact ICI anti-tumor efficacy.

**Methods:**

A pharmacokinetic study of human IgG1 (hIgG1) and hIgG1 with D265A (D265A) mutation, to abrogate all FcγR binding, was performed in mice that were either LLC tumor bearing (TB) or tumor free (TF). Immunofluorescence studies using fluorescence conjugated anti-human IgG were conducted to detect and localize infused hIgG1 in the mouse liver. To further investigate, FcγRIIb knockout mice were utilized in pharmacokinetic studies with hIgG.

**Results:**

CL of both IgG1 and D265A significantly increased in LLC TB mice compared to TF controls, however the CL of D265A was significantly lower compared to hIgG1 in LLC TB mice. Immunofluorescence image of mouse livers portrays colocalization of the administered hIgG1 and FcγRIIb in liver sinusoidal endothelial cells (LSEC), as well as upregulated hepatic expression of FcγRIIb in LLC TB. However, hIgG1 CL was unaffected by whole body knockout of FcγRIIb.

**Conclusion:**

Reduced CL of D265A versus IgG1 in LLC TB mice, but not TF mice, suggests FcγRs are involved in catabolic CL of IgG antibodies in the presence of LLC tumors and cancer cachexia. This suggest that in the presence of LLC tumors, changes in FcγR expression and/or function lead to significantly altered antibody CL mediated by FcγR. This apparent role of FcγRs in antibody catabolism cannot be solely explained by FcγRIIb, but instead suggests the significance of other FcγRs in cachexia-associated increases in antibody CL.

## 1 Introduction

Antibody therapeutics are increasingly the focus of drug development in oncology (1), hallmarked by the introduction of immune checkpoint inhibitors (ICIs). ICIs are a class of antibody drug designed to block inhibitory immune cell signaling in order to stimulate anti-tumor immune responses (2). This novel drug class has displayed improved efficacy over chemotherapy in multiple cancer types, without the off-target toxicities traditionally associated with cytotoxic chemotherapy (3–9). Immune checkpoint inhibitors have become frontline therapy in a monotherapy or dual therapy setting for several cancer types such as non-small cell lung cancer, melanoma, renal cell carcinoma, and more (10–12).

Despite these significant benefits in cancer management, ICIs display significant variability in outcomes, with current estimates suggesting only about 20-40% of patients receiving ICIs show durable responses to therapy, depending on disease type and staging (13–18). There is little understanding of the mechanisms dictating this variation, and major efforts in the field are dedicated to understanding biomarkers and mechanisms of treatment resistance.

Retrospective analyses have identified that an increased rate of ICI clearance (CL), both at baseline (CL_0_) and the magnitude of CL change over time, have been associated with shorter overall survival (OS) in multiple clinical populations such as those with Non-small cell lung cancer (NSCLC), melanoma, urothelial carcinoma, and others (19–25). This observed relationship between ICI CL and efficacy was not remediated by dose increase, and the disconnect between drug exposure and efficacy indicates that ICI CL is not a cause of poor response but instead is a biomarker of therapeutic outcomes (23). Many analyses that have uncovered CL-Response relationships in patients receiving ICIs have also noted that high ICI CL often coincides with markers of cancer-associated cachexia in these patients (23, 25–32). Cancer cachexia is a multifactorial syndrome characterized by irreversible loss of skeletal muscle mass with or without the loss of adipose tissue. This phenotype is the result of a complex interaction between tumor and host factors leading to a chronic state of inflammation, dysmetabolism, and an overall disruption in energy homeostasis (33, 34). Patients with cancer cachexia generally display less benefit from anti-cancer therapy compared to patients without cachexia (35–38), and more specifically, cachexia status in patients correlates with poor outcomes in ICI therapy (23, 28–30). Understanding the relationship between ICI CL, cancer cachexia, and ICI treatment outcomes will have a profound impact upon our understanding of antibody immunotherapy and prognostic indicators of response to ICIs.

We have previously reported that increased ICI CL in correlation with cancer cachexia can be replicated in pre-clinical models. The CL of the anti-human PD-1 IgG4 pembrolizumab was elevated in the well-established Lewis lung carcinoma (LLC) and colon-26 (C26) murine models of cancer cachexia, but not in the non-cachectic MC38 tumor model (39, 40). This finding and other analyses highlight how increased ICI CL cannot be explained solely by the presence of a tumor/cancer alone (39). Interestingly, pembrolizumab has no affinity for murine PD-1, thus these observed changes in CL cannot be attributed to target binding and subsequent target-mediated drug disposition, but rather suggests cachectic-dependent changes in catabolic CL. However, the Fc portion of pembrolizumab retains affinity for murine Fc receptors (FcR) leaving open the possible involvement of these receptors in ICI disposition in pre-clinical models.

The FcRs that bind and interact with IgG are the neonatal Fc receptor (FcRn) and the Fc-γ receptors (FcγRs). FcRn is a predominantly intracellular receptor that is expressed in immune cells and certain endothelial tissues. Binding of the Fc portion of an antibody to FcRn protects IgG from lysosomal degradation, and recycles the antibody back to the cell surface (41). Interaction of the Fc portion of antibodies with FcRn can have a drastic impact on the pharmacokinetics (PK) of ICIs. However, our previous work investigating FcRn’s role in cancer cachexia-associated increases in ICI CL using an antibody with reduced FcRn binding suggested potential contributions of non FcRn-mediated catabolic pathways, such as FcγRs (39).

Murine FcγRs are expressed mainly on immune cell surfaces and are comprised of the activating receptors FcγRI, FcγRIII, and FcγRIV and the sole inhibitory receptor FcγRIIb (RIIb) (42, 43). Binding of the Fc portion of the antibody to activating receptors leads to Fc effector functions, such as antibody dependent cellular phagocytosis (ADCP), antibody dependent cellular cytotoxicity (ADCC), cytokine secretion, cellular activation, and more (44–47). Antibody Fc binding to RIIb leads to inhibition and regulation of activating FcγR effector function, although it has been reported that certain isoforms of RIIb can perform endocytosis functions (42, 48, 49). It is understood from previous research in the context of autoimmunity, that transient expression of these receptors, both outright and in relation to one another, can play a role in disease pathogenesis and severity (43, 50, 51). With the introduction of immunotherapy, these receptors have become a focus in the field of oncology due to their ability to profoundly impact antibody based therapeutics, especially in the case of anti PD-1 drugs. In preclinical models, it has been understood that the affinity of Fc portion of antibody for FcγRs can vastly affect anti-tumor outcomes in anti PD-1 therapy. Optimal anti-tumor efficacy in anti PD-1 therapy is seen in antibodies with the least affinity for FcγRs, likely due to mitigation of FcγR-mediated T cell depletion pathways (48, 52, 53). Clinical anti-PD-1 drugs are built upon a human IgG4 background which is known to have low, but non-negligible, affinity for FcγRs (54). Our group has previously demonstrated that FcγR expression on immune cells is altered in correlation with cancer cachexia and skeletal muscle wasting in pre-clinical and clinical populations, highlighting how disease state can induce aberrant FcγR expression (55).

This study was conducted to understand how cancer cachexia-associated changes in FcγRs contribute to increased ICI CL in pre-clinical murine models. Given FcγRs clear role in the efficacy of ICI therapy, understanding FcγR contributions to cachexia-associated increases in antibody CL is imperative. Additionally, previous studies have demonstrated that the liver is a major organ involved in the catabolism of IgG (56) and even further, IgG mutants with reduced FcγR affinity showed drastically reduced IgG accumulation and deposition in the liver, suggesting the importance of liver FcγRs in antibody PK (57). RIIb is the most abundantly expressed FcγR in the liver, and is predominantly expressed on liver sinusoidal endothelial cells (LSECs) (49). The two isoforms of RIIb, FcγRIIB1 (B1) and FcγRIIB2 (B2) are attributed to regulatory signaling and endocytosis, respectively (58). RIIb on LSECs is of the B2 isoform and is highly endocytic, as best demonstrated by its role in facilitating the CL of small immune complexes from hepatic arterial flow (49). Importantly, RIIb has been shown to impact anti-tumor efficacy of ICI treatment (48, 53, 59, 60). Consequently, this study is aimed to understand the role of FcγR, and specifically liver RIIb, in cachexia associated increases in ICI CL.

## 2 Methods

### 2.1 Animals

FcγRIIb knock-out mice in C57BL/6 background (RIIb -/-) were a gift to LPG via Dr. Clark Anderson (Ohio State University) and from Dr. Jeffrey Ravetch (Rockefeller University). Wild-type C57BL/6J mice that were age and sex matched with RIIb -/- were obtained from The Jackson Laboratory (Bar Harbor, ME) and acclimated for a minimum of 3 days after arrival. Euthanasia occurred by asphyxiation with CO_2_ followed by exsanguination. All animal studies were performed in accordance with protocols approved by the Institutional Animal Care and Use Committee at The Ohio State University (protocol # 2017A00000117), and with responsibilities and procedures outlined in The Ohio State University’s Animal Welfare Assurance (D16–00168 [A3261–01]) as approved by the U.S. Public Health Service Office of Laboratory Animal Welfare.

### 2.2 Cell culture

LLC (ATCC, Manassas, Virginia) and MC38 (Kerafast, Boston, MA) cells were cultured at 37 °C in a humidified chamber with 5% CO_2_ in Dulbecco’s Modified Eagle Medium (Invitrogen, Waltham, MA) supplemented with 1% penicillin-streptomycin (Alkali Scientific, Ft. Lauderdale, FL) and 10% fetal bovine serum (Biowest, Riverside, MO). For injection into mice, cells were harvested (LLC: Passages 8-10, MC38: Passages 4-6) by trypsinization followed by centrifugation to pellet the cells in growth medium, after which the cells were resuspended in sterile phosphate buffer saline (PBS) to a concentration of 10.0 x 10^6^ cells/mL. LLC cells were confirmed negative for mycoplasma by Plasmotest kit (Invivogen, San Diego, CA).

### 2.3 *In vivo* tumor studies

The LLC and MC38 tumor models were conducted as previously described (39, 40). Briefly, all mice were group-housed under constant photoperiod conditions (12-h light/12-h dark) with *ad libitum* access to water and standard diet. Mice included in the study were randomly assigned to tumor-free (TF) or LLC/MC38 tumor-bearing (TB) groups at 8–12 weeks of age. On day 0, mice were briefly anesthetized (3% isoflurane) and injected with 50 μl of either PBS (TF) or 0.5 x 10^6^ LLC cells in PBS (TB) into the biceps femoris muscle of the right hind limb or 100 µl of 0.5 x 10^6^ MC38 cells in PBS (TB) subcutaneously in the right flank. Mouse body weights and tumor volume measurements were recorded at minimum once a week. Tumor size was measured using digital calipers and tumor volume was calculated using a standard formula l x w x h x π/6 for intramuscular tumors or l x w^2^ x π/6 for subcutaneous tumors. Tumor volume was converted to an estimated mass (1 cm^3^ = 1 g) in order to derive tumor-adjusted body weights during the course of the study. At euthanasia, terminal excised tumor mass was subtracted from terminal body weight to derive tumor-adjusted body weight. For CO_2_ euthanasia, the CO_2_ flow rate is set to ensure a 30%-70% displacement of the chamber volume/min. For PK studies investigating human IgG1 D265A mutant, a total of two independent studies were completed. For RIIb -/- PK investigations, a total of three independent studies were completed.

### 2.4 Euthanasia and tissue collection

Mice were euthanized by CO_2_ asphyxiation followed by exsanguination by cardiac puncture. Terminal blood was collected into heparinized tubes with gel separator (BD Biosciences, Franklin Lakes, NJ, USA) and subsequently centrifuged at 10,000 x g for 2 min. Plasma was then collected and stored at -80°C until analysis. At euthanasia, terminal bodyweights were recorded, and the following tissues were resected and weighed: gastrocnemius, tibialis anterior, quadriceps muscles from left hind limb, epididymal (from males) or parametrial (from females) adipose tissue, liver, spleen, and tumor.

### 2.5 Pharmacokinetic studies

Murine pharmacokinetic (PK) studies were carried out as previously described (39, 40, 61). On day 14 post-tumor inoculation, mice were administered a 100 µl single intravenous dose of either human IgG1 (200 μg or 100 μg; Cat# C0001, MBL Life Science, Woburn, MA, USA) or human IgG1 D265A (200 μg; Cat# C0020, MBL Life Science). Notably, these monoclonal human IgG1 and D265A antibodies used in this study bind specifically to hen egg lysozyme, which is not present in mice, and therefore with single doses, CL of these antibodies is via non-specific, catabolic processes. Serial blood samples were collected into heparinized tubes at 1, 24, 48, 96, 144, 192, and 240 h after antibody administration, centrifuged at 2000 x g for 5 min, and plasma supernatant collected and stored at -80°C until analysis. Mice displaying apparent absorption phases were excluded from analysis. Terminal samples were collected by cardiac puncture at euthanasia. All blood samples were centrifuged after collection, from which the plasma supernatant was harvested and stored at -80°C until further analysis. For studies investigating IgG1 pharmacokinetics in WT and RIIb -/- mice, plasma concentration versus time curves were plotted as % of initial dose, calculated as [Plasma IgG1 concentration (ug)/Initial Dose (200μg or 100μg) * 100].

### 2.6 Antibody measurement by ELISA

Free, unbound, human IgG1was measured by ELISA as described (39, 40). Briefly 100 μl/well of Hen Lysozyme Antigen (Cat# 10837059001, Roche, Indianapolis, USA) at a concentration of 3 μg/mL was allowed to incubate overnight at 4°C in a transparent, clear bottom, high-binding 96 well plate (NEST, Rahway, NJ). Wash steps were completed 3 times each at a volume of 300 μl/well. All samples and standards were loaded at 100 μl/well. Incubation was performed on a plate shaker with gentle orbital shaking (300 rpm). Standard concentrations ranged from 5–500 ng/mL, and all samples were run in duplicate. Samples were diluted either 400x or 800x in blocking buffer (PBS, 0.05% Tween-20, 1% bovine serum albumin). Anti-human IgG1-HRP conjugate (Cat# A10648, Invitrogen, Carlsbad, CA, USA) secondary detection antibody was added at a volume of 100 μl/well and at a concentration of 0.2 μg/mL. Absorbance was measured using a Biotek Synergy H1 plate reader (Agilent, Santa Clara, CA, USA) at 450 nm corrected at 570 nm.

### 2.7 Pharmacokinetic parameter estimation

Nonlinear mixed effect modeling in NONMEM version 7.3 (Icon, Dublin, Ireland, Version 7.3) was used to estimate PK parameters from plasma human IgG1 concentrations in mice as described previously (40, 62). GraphPad (v9.4, GraphPad Software, San Diego, CA) was used for visual representation of data. Human IgG1 and IgG1 D265A concentrations were fit to a linear, intravenous, 2 compartment model, as described previously (39, 40) to derive individual PK parameter estimations. The model was parameterized with clearance (CL), volume of distribution of the central compartment (V1), inter compartmental clearance (Q), and volume of distribution of the peripheral compartment (V2). Additionally, an exponential error model was used in order to describe inter individual variability (IIV), and an additive error model for log transformed data was used to model the residual variability (ε).

### 2.8 Immunofluorescence

Immunofluorescence was performed as previously described (61, 63). A section of the left lobe of the liver was placed into a plastic cassette and incubated in 4% paraformaldehyde (ThermoFisher, Waltham, MA, USA) for 2 h at room temperature, followed by consecutive overnight incubations in PBS containing 0.01% sodium azide (PBS+Azide) and then PBS+Azide containing 20% sucrose. The tissue section was then patted dry and completely submerged into a plastic tissue base mold (ThermoFisher) containing OCT Tissue Freezing Media (Electron Microscopy Sciences, Hatfield, PA, USA) and stored at -80°C until ready for sectioning. Five-µm thick sections were cut from the frozen tissue blocks and positioned onto glass slides (Fisherbrand™ Superfrost™, ThermoFisher) and stored at -80°C. For staining, sections were hydrated for 15 min in PBS+Azide, blocked for 1 h in PBS+Azide containing 5% non-fat milk powder (Bio-Rad, Hercules, CA, USA), and then washed 3-times with PBS+Azide. Sections were incubated overnight with primary antibodies: 1:25 dilution of rabbit anti-mouse FcγRIIb (Clone: AT130-5, Novus Bio, Centennial CO, USA), and 1:25 dilution of rat anti-mouse F4/80 (Clone: A3-1, Bio-Rad) in blocking buffer and then washed 3 times. Secondary antibodies, goat anti rabbit 568 (ThermoFisher), goat anti rat 680 (ThermoFisher), and donkey anti human 488 (Jackson Immuno-Research, West Grove, PA, USA), were diluted 1:100 in blocking buffer, added to sections, and allowed to incubate for 1h protected from light. After vigorous washing with PBS+Azide every 15 min for 1h, sections were incubated with DAPI (Sigma-Aldrich) for 10 min and then washed again. Coverslips were mounted with ProLong Gold mounting media (ThermoFisher). Images were taken using the 40x objective on an Olympus FV1000 confocal microscope (Olympus Life Sciences, Waltham, MA, USA).

### 2.9 Immunoblot

Immunoblot was conducted on liver tissue lysates from representative animals as described previously (64). Briefly, liver tissue was lysed using an in-house lysis buffer (25 mM HEPES, 20mM Na_4_H_2_O_7_, 100mM NaF, 4mM EDTA, 2mM Na_3_VO_4_, 1% Triton X-100, 0.34 mg/mL PMSF, 0.01 mg/mL aprotinin, and 0.01 mg/mL leupeptin) and mechanically homogenized by glass homogenizer. Equivalent amounts of protein from each tissue lysate were determined via Bradford assay (Bio-Rad), then resolved by SDS-PAGE. Proteins were transferred onto nitrocellulose (Bio-Rad) membrane by semi-dry transfer. Membranes were then quickly washed with TBST (Tris-Buffer Saline + 0.1% Tween-20) and then incubated for 1 h with gentle rocking at room temperature in TBST + 5% bovine serum albumin (Alkali Scientific, Ft. Lauderdale, FL, USA) for blocking. After quickly washing with TBST, membranes were incubated overnight at 4 °C with gentle rocking with primary rabbit anti-mouse RIIb serum (generous gift from Dr. John Cambier) at a dilution of 1:2000 or mouse anti-GAPDH (Santa Cruz Biotechnology, Dallas, TX, USA). Membranes were washed 3 times with TBST and then incubated with the HRP-conjugated donkey anti-rabbit IgG or rabbit anti-mouse IgG secondary antibody at a dilution of 1:5000 for 1 h at room temperature. Membranes were then washed 3 times with TBST and visualized with ECL chemiluminescence (ThermoFisher). Relative Immunoblot quantification was performed as previously described (65) using Fuji Image J (66).

### 2.10 Statistics

Phenotypic results (body weight, tissue mass, etc.) from mice in the human IgG1 D265A mutant studies were analyzed by Mann-Whitney test like previously described investigations (39, 40). Body weight data from mice enrolled in the RIIb -/- PK studies were log2-transformed to minimize variations but represented in original scale. Analysis of variance (ANOVA) followed by pairwise comparisons between groups was performed, and data normality assumptions were affirmed using residual plots of models. *Post-hoc* pairwise tests were adjusted for multiple comparisons using the Hochberg method. Additionally, body and tissue mass data from FcγRIIb -/- mouse studies were analyzed by two-way ANOVA, using genotype and tumor status as covariates to evaluate the interaction between factors. Comparisons of CL rates between groups were tested by Wilcoxon Signed-Rank test. Analyses were carried out using GraphPad (GraphPad Software), and R Studio (R Core Team, Vienna, Austria).

## 3 Results

### 3.1 FcγRs contribute to antibody catabolism in the LLC murine model of cancer cachexia

**Figure 1:**
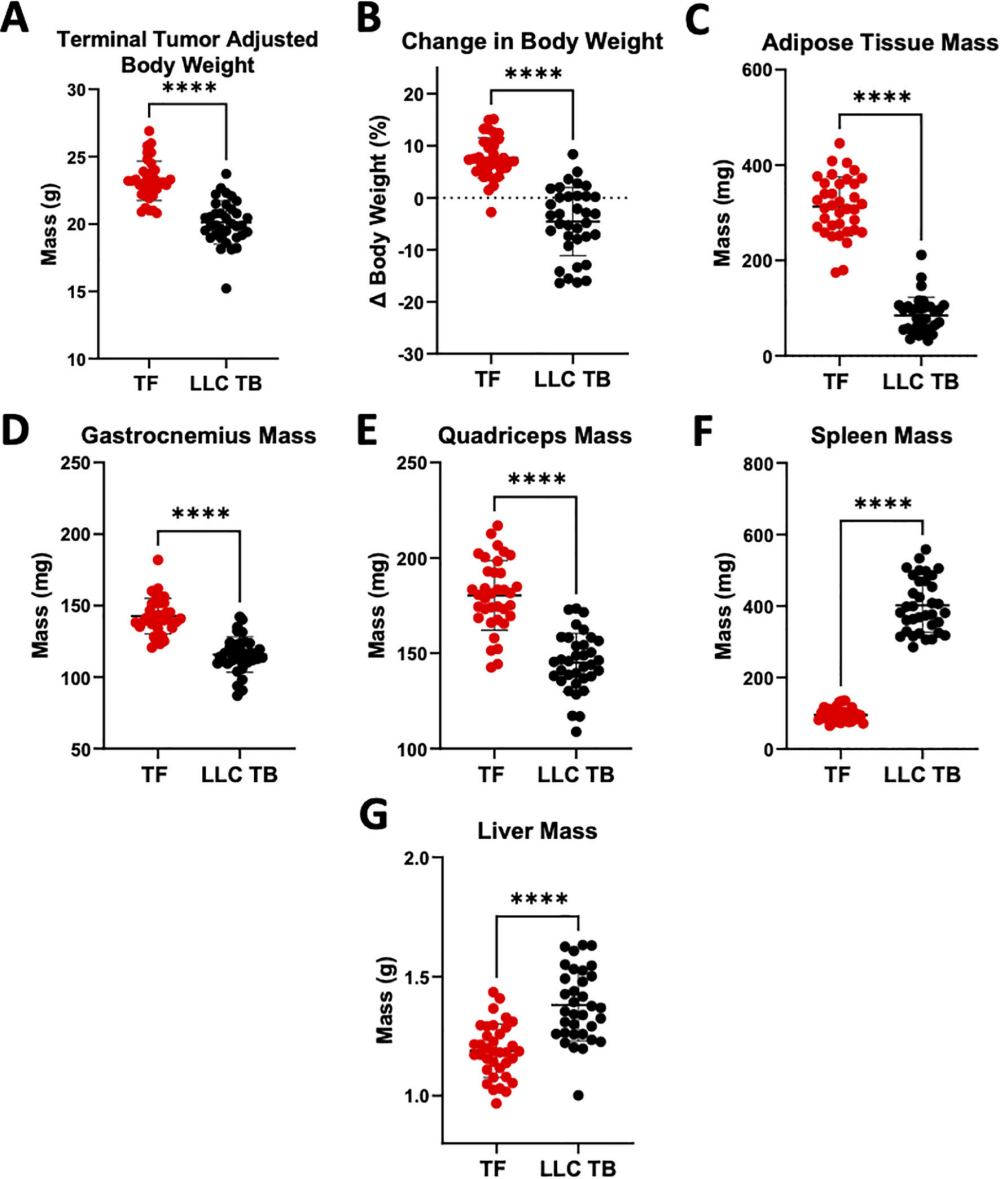
Mice with Lewis Lung Carcinoma (LLC) Tumors display Phenotypic effects of Cancer Associated Cachexia. Male Tumor Free (TF) C57BL/6J mice (n=36) or mice with Lewis lung carcinoma (LLC) tumors (n=35) were inoculated on Day 0 with either PBS or LLC cells respectively. Euthanasia at end of study (EOS) occurred on either day 24 (Study 1) or day 22 (Study 2) and terminal body weight measurements were recorded for analysis of tumor induced cachexia effects. At study endpoint **(A)** Tumor adjusted terminal bodyweight (total mouse weight at EOS minus the observed tumor mass) **(B)** Tumor adjusted terminal bodyweight change, expressed as a percentage of initial (day-1) bodyweight, **(C)** Epididymal adipose tissue mass, **(D)** Gastrocnemius mass, **(E)** Quadriceps mass, **(F)** Spleen mass, **(G)** Liver mass. Mean ± SD; ****P <0.0001 by Mann-Whitney.

In order to understand FcγR involvement in cancer cachexia-associated antibody catabolism, a PK study was conducted using human IgG1 (hIgG1) and human IgG1 with the D265A mutation (D265A), which abrogates affinity to all four classes of murine FcγRs and complement receptors (67). A total of 36 healthy C57BL/6J (TF) mice and 35 mice bearing LLC tumors (LLC TB) were enrolled in two separate experiments. At end of study, LLC tumor-bearing mice displayed a cachectic phenotype that included decreased terminal tumor-adjusted body weight (**Figure 1A**), % change in adjusted body weight over study timeline (**Figure 1B**), adipose tissue mass (**Figure 1C**), gastrocnemius mass (**Figure 1D**), and quadriceps mass (**Figure 1E**), but significant increases in spleen (**Figure 1F**) and liver mass (**Figure 1G**) versus TF mice.

**Figure 2:**
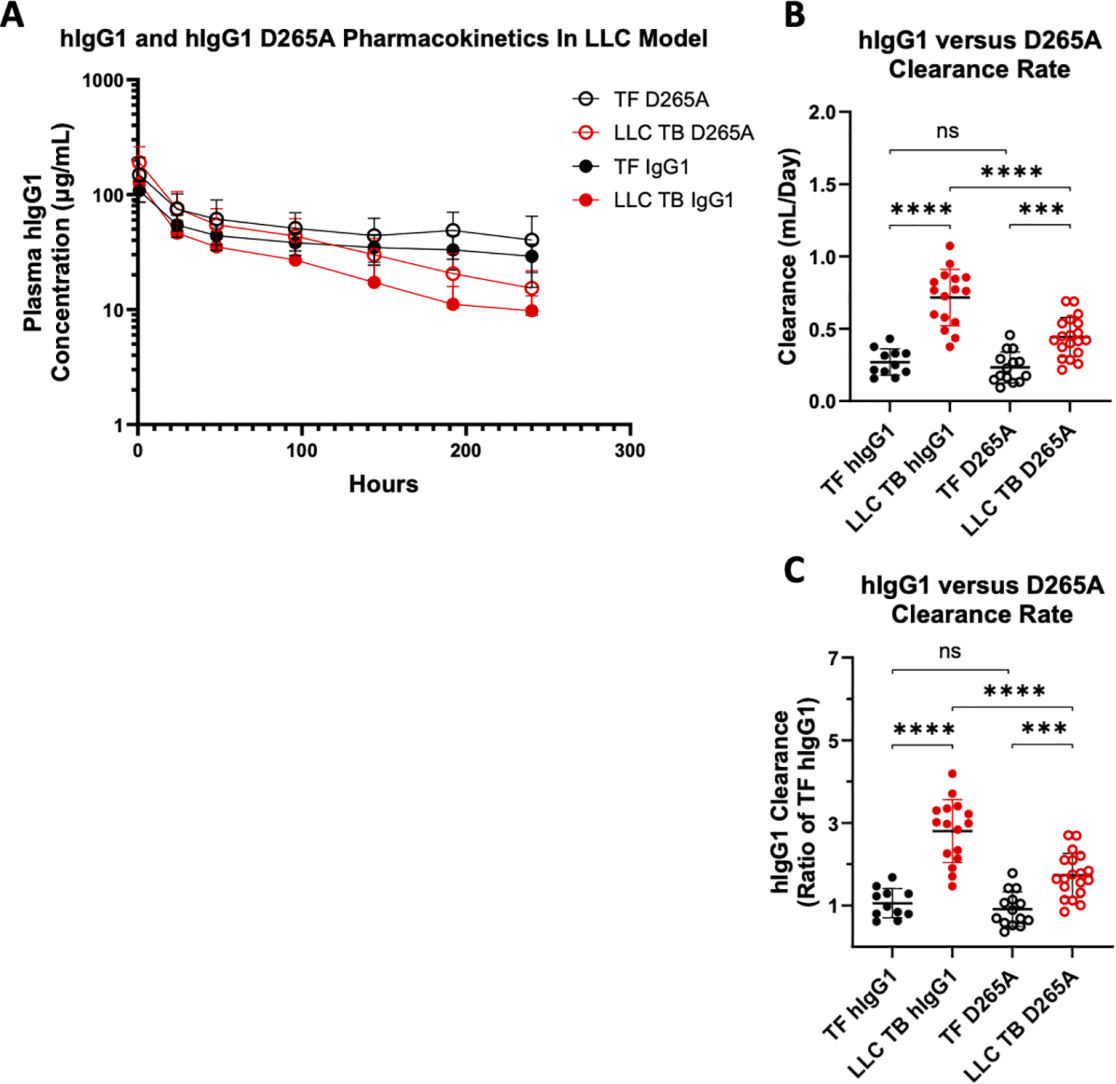
D265A antibody clearance is reduced when comparing LLC TB mice. 8-10 week old male C57BL/6J mice were injected with either Lewis Lung Carcinoma cells (LLC; n=35) or PBS (TF; n=36) day 0. On day 14, available mice were administered a single 200 μg intravenous injection of either hIgG1 (n=27), or hIgG1 D265A (n=33) with serial bleeds at 1, 24, 48, 96, 144, 168, 192, and 240h until end of study (EOS). Pharmacokinetic plots of hIgG1 and D265A illustrating **(A)** Plasma concentration of hIgG1 and D265A in TF/LLC TB mice and **(B)** Individual antibody clearance (CL) estimates (Mean ± SD) and **(C)** Antibody clearance estimates normalized to TF hIgG1 control (Geometric Mean + geometric SD) across groups of TF hIgG1 (n=11), LLC TB hIgG1 (n=16), TF D265A (n=14), and LLC TB D265A (n=19); ***P < 0.001; ****P <0.0001 by Wilcoxin Signed-Rank test.

At day 14 post-tumor cell injection, mice were administered 200 µg of either hIgG1 (n=29) or D265A (n=35), intravenously. There was no statistical difference in tumor mass (p=0.48 by Mann-Whitney) between TB mice receiving hIgG1 (5.02g ± 0.79) or D265A (5.18g ± 0.98) administrations. A total of 7 plasma PK timepoints per mouse were collected and used in PK parameter estimation. Mice displaying an apparent absorption phase arising from errant extravascular dosing were excluded from the analysis. Plasma concentration versus time profiles of hIgG1 and D265A antibodies from TF and LLC TB mice are displayed in **Figure 2A**. **Figure 2B** illustrates individual antibody CL estimates across groups in which both hIgG1 and D265A displayed increased CL as a function of tumor status, as CL in both LLC TB groups were significantly elevated relative to their respective TF controls (**Figure 2B**). **Figure 2C** depicts individual clearance estimates as normalized to TF hIgG1 control group. Interestingly, the CL of D265A in TB mice was significantly lower than that of hIgG1 antibody in TB mice (p<0.0001). Specifically, the average CL of hIgG1 was 0.716 (± 0.091) mL/day compared to an average D265A CL of 0.442 (± 0.136) mL/day in LLC TB mice. This difference in CL between the two antibodies, however, was not observed in TF mice. These findings suggest that FcγR binding contributes to the observed increases in CL in the context of cancer cachexia, but not under normal physiological conditions. Nonetheless, the increased CL of D265A in TB mice relative to TF mice implies that cachexia-associated increases in catabolic CL are not solely mediated through FcγRs, and that other mechanisms significantly contribute to increased CL in cachectic TB mice. There was no impact of study as a factor on pharmacokinetic estimates in this investigation.

### 3.2 Liver FcγRIIb colocalizes with IgG1 and is increased in LLC TB mice

**Figure 3:**
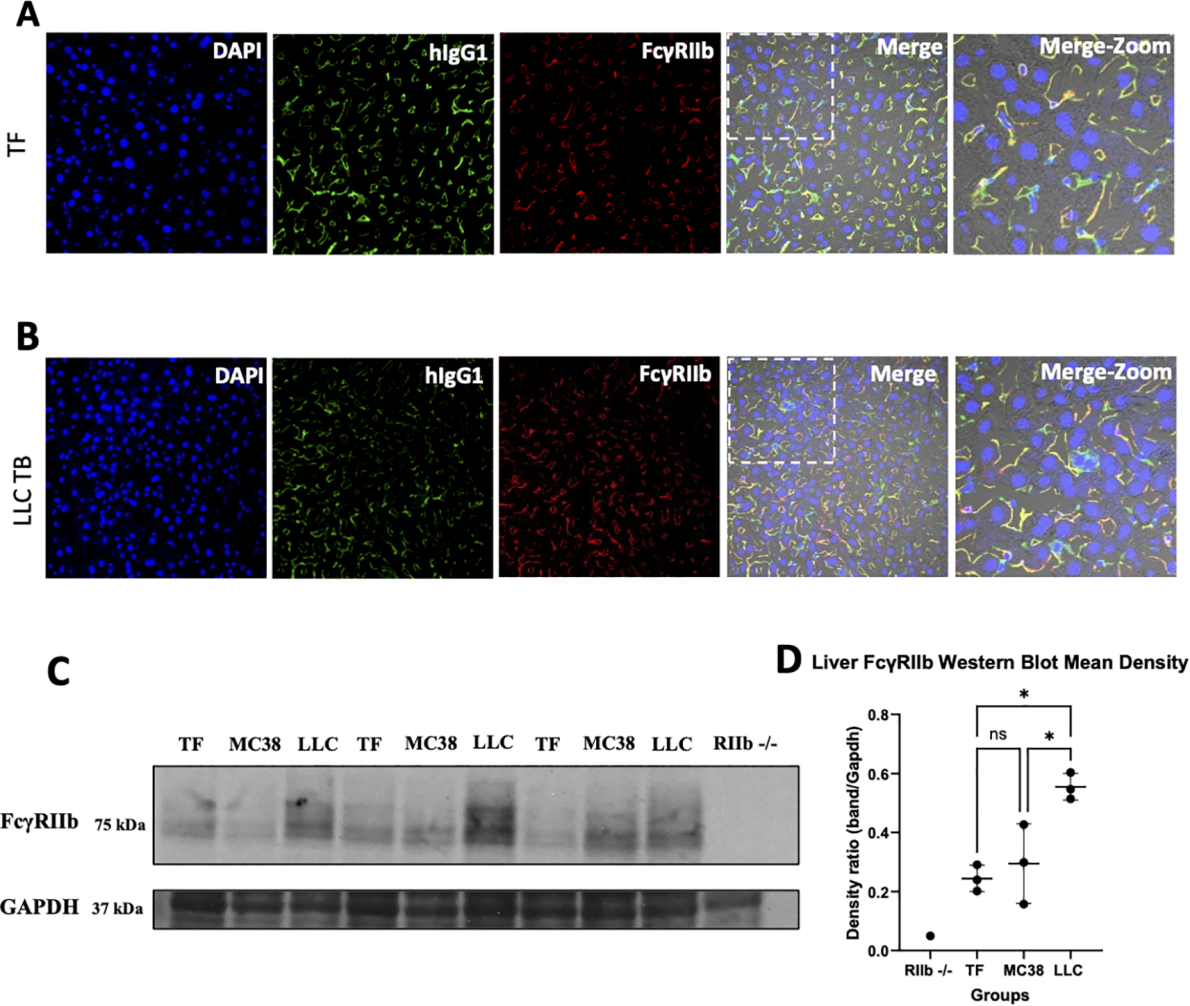
Liver FcyRIIb colocalizes with intravenously administered human IgG1 and the expression of liver FcyRIIb expression is upregulated in LLC model of cancer cachexia. Immunofluorescent imaging of mouse liver 24h post intravenous injection of hIgG1 at 40x objective illustrating the colocalization of injected hIgG1 (green) and FcyRIIb (red) in male **(A)** Tumor Free and **(B)** LLC Tumor bearing mice. The last panel represents a higher-magnification view of the merged image shown in the preceding panel, indicated by the dotted white outline. **(C)** Western blot illustrating changes in FcyRIIb liver expression across TF (n=3), MC38 (n=3), and LLC (n=3) tumor bearing mice. **(D)** Western blot band density estimates normalized to GAPDH Mean ± SD; *P < 0.05 by One-way ANOVA followed by Tukey's multiple comparison with Hochberg correction.

The liver is a major site of antibody catabolism, abrogating FcγR binding reduces antibody accumulation in liver (56, 57) and FcγRIIb is abundantly expressed in liver, specifically in liver sinusoidal endothelial cells (LSECs) (49). To understand the role of hepatic FcγRIIb in IgG CL and catabolism, we used immunofluorescence staining to examine the localization of infused hIgG1 relative to FcγRIIb within the livers of mice 24 hours post-administration in TF and cachectic LLC TB mice. ​ As shown in **Figures 3A, B**, infused hIgG1 (green), stained with Alexa Fluor-488-conjugated anti-human IgG antibody, colocalized with FcγRIIb (red) in the liver sinusoids, as evidenced by the yellow overlap in the merged images, regardless of tumor status. To evaluate the specificity of the staining protocol, parallel liver sections were stained with anti-human IgM conjugated to FITC. These control experiments revealed no detectable fluorescence, confirming the absence of non-specific binding by the secondary antibody (**Supplementary Figure 1A**). Since we have identified previously (55) that FcR expression on immune cells is modulated in correlation with cancer cachexia, we hypothesized that there were changes in liver endothelial cell FcγRIIb expression in LLC TB mice. To address this question, the abundance of FcγRIIb protein was assessed by immunoblot on the liver tissue lysates from TF, LLC TB mice, and mice bearing non-cachectic MC38 tumors (MC38 TB), which do not display elevated ICI CL and served as a TB control (39), and RIIb-/- mice as control. All mice were naïve to exogenous IgG exposure. As expected, no FcγRIIb protein was detected in the FcγRIIb -/- control mice (**Figure 3C**), while FcγRIIb expression was approximately 2-fold higher in LLC TB mouse livers than in those of TF and MC38 mice (**Figure 3D**). Considering the importance of liver as a site of antibody catabolism, the observed colocalization of exogenous hIgG1 and liver FcγRIIb on LSECs and the apparent elevation of liver FcγRIIb expression in the LLC model of cancer cachexia suggest the involvement of liver FcγRIIb in cancer cachexia-associated increases in antibody CL.

### 3.3 Deletion of FcγRIIb does not affect cancer cachexia-associated elevation in IgG1 clearance

**Figure 4:**
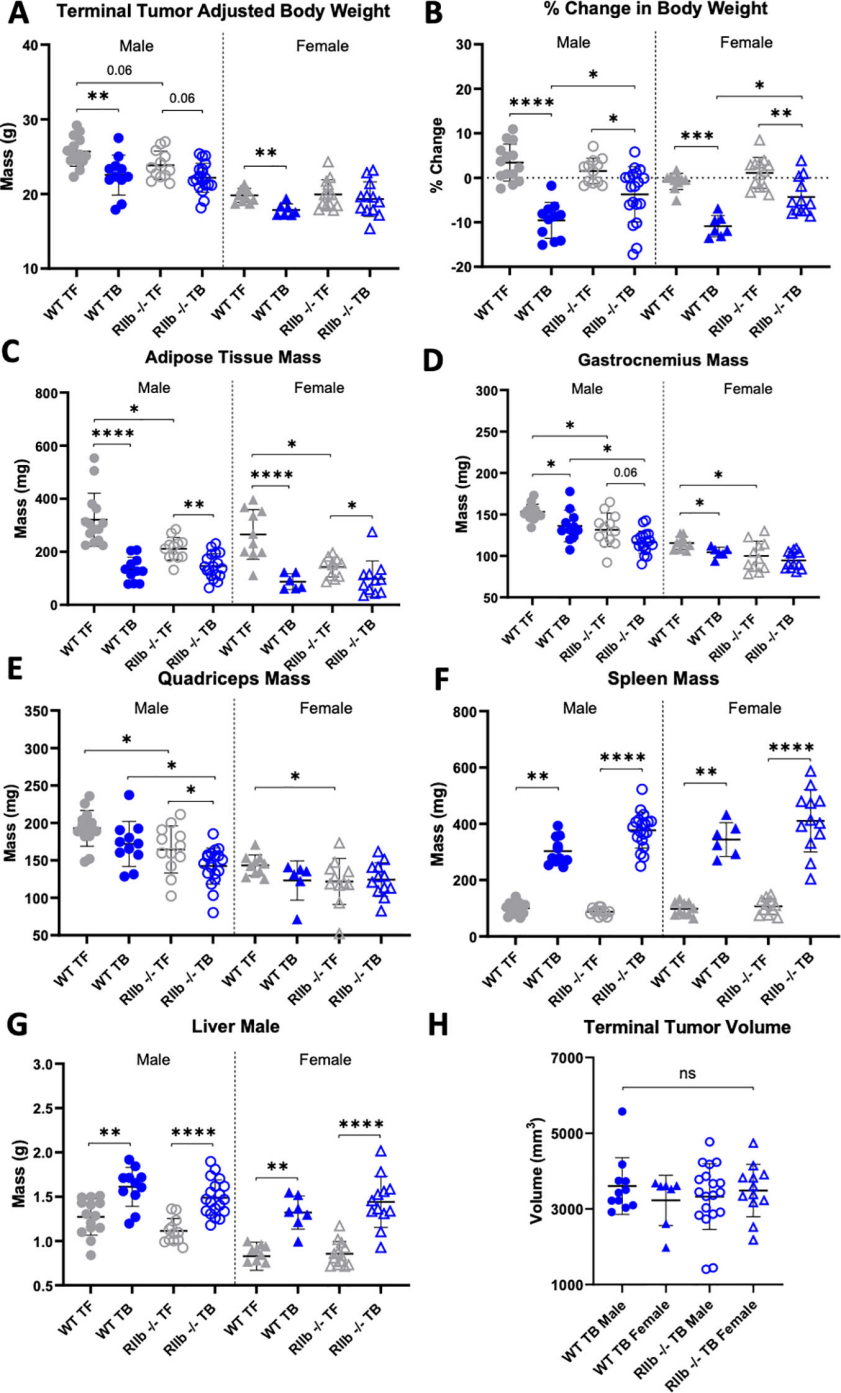
LLC tumors induce cachectic phenotype FcγRIIb -/- mice. On day 0, male and female wildtype (WT; n=42) or FcγRIIb -/- (RIIb -/-; n=57) mice were inoculated with either LLC cells (n=50), or PBS for tumor free mice (n=49). Study was terminated on day 22 post inoculation and mouse body weight and tissue mass was recorded for analysis of tumor-induced cachexia effects. Mouse body weight data was separated based on male (n=56) and female (n=43). At study endpoint **(A)** Tumor-adjusted terminal bodyweight, total mouse weight at EOS minus the observed tumor mass **(B)** Tumor adjusted terminal body weight change, expressed as a percentage of initial (day-1) body weight, **(C)** Adipose tissue mass, **(D)** Gastrocnemius mass, **(E)** Quadriceps mass, **(F)** Spleen mass, **(G)** Liver mass and **(H)** Terminal tumor volume. Mean ± SD; *P < 0.05 **; P < 0.01; ***P < 0.001; ****P < 0.0001 by Tukey’s Multiple Comparison test with Hochberg correction.

To investigate the role of FcγRIIb in cancer cachexia-associated increases in hIgG1 CL, a PK study was performed in wild type C57BL/6J mice (WT) and mice with global (whole body) knockout of FcγRIIb (RIIb -/-) harboring LLC tumors. A total of 99 mice were enrolled in three separate studies that were stratified by tumor status (TF, n=49; TB, n=50), sex (male, n=56; female, n=43), and genotype (WT, n=42; RIIb -/-, n=57), all receiving a single intravenous dose of either 200 µg (Study 1, n=15) or 100 µg (Studies 2 and 3, n=84) of hIgG1 on day 14 post-tumor cell injection. In order to test if FcγRIIb knockout impacted LLC-induced changes in body weight, a two-way ANOVA was performed to test the effects of tumor status and genotype on cachectic endpoints in male and female mice (**Table 1**). A significant interaction between genotype and tumor (e.g. p<0.05 in the genotype:tumor columns of **Table 1**) would suggest that genotype significantly affects the magnitude of change in body weight endpoints between TF and LLC TB mice potentially confounding our interpretation of hIgG1 PK in cachectic RIIb -/- mice. Body weight and terminal tissue data are plotted in **Figure 4**, and statistical differences between the means of groups were illustrated by multiple comparison pairwise tests. **Table 1** shows, in male mice, that genotype impacted adipose tissue and spleen mass changes differentially between TF and TB groups. Likewise, female mice displayed a significant interaction in % change in adjusted bodyweight, suggesting that female RIIb -/- mice did not exhibit the same magnitude of body weight loss over the course of the study as WT controls.

**Table 1:** Knockout of FcyRIIb in mice does not significantly alter LLC-induced cachexia phenotype.

p values	Male			Female		
	Genotype	Tumor	Genotype: Tumor	Genotype	Tumor	Genotype: Tumor
Adjusted BW	0.0175	0.0002	0.2285	0.2982	0.0286	0.2219
% Change in Adjusted BW	0.5073	0.0034	0.6961	0.0016	<0.0001	0.0282
Adipose Tissue (g)	0.0036	<0.0001	0.0044	0.0089	<0.0001	0.0559
Liver (g)	0.1579	<0.0001	0.5415	0.1291	<0.0001	0.7838
Spleen (g)	<0.0001	<0.0001	0.0014	0.0088	<0.0001	0.8271
Gastroc (g)	<0.0001	0.0014	0.7842	0.0025	0.0266	0.5956
Quadriceps (g)	0.0068	0.0028	0.9439	0.0103	0.0341	0.1628

2- way ANOVA results on male (n=56) and female (n=43) mouse body weight (BW) using factors of genotype (WT, n=42; RIIb -/-, n=57), and tumor status (TF, n=49; TB, n=50) to test the impact of each factor both independently, and in relation to each other, on cachexia endpoints in mice. A total of n=99 mice were included in the analysis.

As expected, LLC tumor status was a significant factor upon tumor-adjusted terminal body weight (**Figure 4A**), % change in adjusted terminal body weight (**Figure 4B**), adipose tissue mass (**Figure 4C**), gastrocnemius mass (**Figure 4D**), quadriceps mass (**Figure 4E**), spleen mass (**Figure 4F**), and liver mass (**Figure 4G**). Interestingly, RIIb -/- mice, irrespective of tumor, exhibited significantly decreased gastrocnemius and quadriceps mass (**Figures 4D, E**) in both male and female mice (genotype columns, **Table 1**). Importantly, there were no significant differences in terminal tumor volume across groups (**Figure 4H**), indicating that terminal tumor burden was not significantly affected by whole body RIIb knockout.

**Figure 5:**
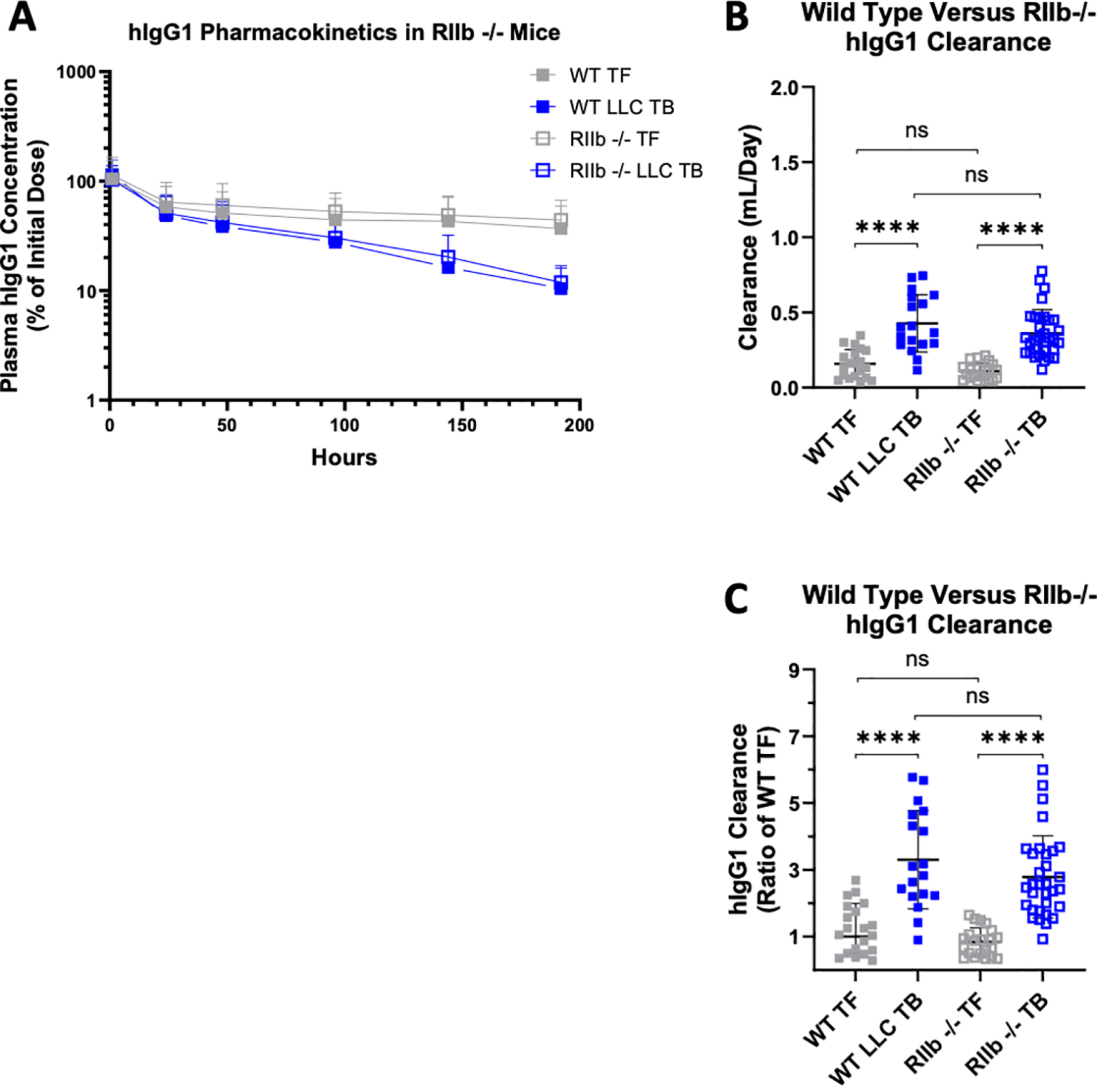
hIgG1 pharmacokinetics are unaffected by knockout of FcyRIIb in TF and LLC TB mice. 8-14- week-old male and female C57BL/6 mice (WT) or C57BL/6 FcyRIIb -/- (RIIb -/-) were given an injection of either PBS (TF) or LLC cells (LLC TB) on day 0 to make the 4 groups of WT TF (n=19) WT LLC TB (n=18), RIIb -/- TF (n=21) and RIIb -/- LLC TB (n=31). On day 14, mice were administered a single intravenous dose of 200 μg (Study 1; n=15) or 100 μg (Study 2 + 3; n=84) hIgG1 with serial bleeds at 1, 24, 48, 96, 144, 168, and 192h until EOS. **(A)** Pharmacokinetic plots of hIgG1 plasma concentration in WT or RIIb -/- mice with and without LLC tumors illustrated as % of initial dose [Plasma hIgG1 concentration/Initial Dose (100 or 200 ug) * 100]. **(B)** Individual antibody clearance estimates (Mean ± SD) and **(C)** Antibody clearance estimates normalized to WT TF control (Geometric Mean ± geometric SD) across groups; ****P < 0.0001 by Wilcoxin Signed-Rank test.

**Figure 5A** depicts the plasma concentration of IgG1 versus time in WT and RIIb-/- mice with and without LLC tumors. When comparing the individual CL estimates of hIgG1 (**Figure 5B**), and individual CL estimates normalized to WT TF control group (**Figure 5C**), there was a significant elevation in CL as a function of tumor status in both WT and RIIb -/-, agreeing with previously published studies (39, 40, 61). Congruent with the D265A PK data where FcyR binding was abrogated (**Figures 2B, C**), there was no effect of RIIb expression status on the CL of hIgG1 in TF mice, as WT and RIIb-/- hIgG1 CL was not significantly altered. However, when comparing LLC TB groups, there was no difference in the CL of hIgG1 between WT and RIIb -/- mice (p=0.26). This would suggest that there was no effect of whole body RIIb knockout on the magnitude of hIgG1 CL increase in the LLC model of cachexia, differing from what was observed with the D265A antibody. In other words, RIIb knockout had no effect on cancer cachexia-associated increases in catabolic CL. Additionally, this investigation was completed over the course of three different studies utilizing both male and female mice, as well as different IV dose levels of hIgG1 (200 µg in Experiment 1; 100 µg in Experiment 2). Neither sex (**Supplementary Figure 2A**) nor dose of hIgG1 (**Supplementary Figures 2B–D**) significantly impacted CL rates of hIgG1 in this study.

## 4 Discussion

Uncovering mechanistic pathways underlying the relationship between CL and response regarding ICIs should be of paramount interest to the field of cancer immunology and pharmacology. It is understood that patients with cancer cachexia display increased ICI CL and this correlates with poor therapeutic outcomes independent of drug exposure (23, 26–30, 32). In murine models of cancer cachexia, we see elevated CL of hIgG4 pembrolizumab, but this increased CL is absent in the non-cachectic MC38 model, suggesting increased CL is not a function of tumor status alone (39, 40). Given the importance of FcγRs in ICI efficacy (52, 53) and their suggested role in antibody CL (48, 68), we investigated FcγRs’ contribution to cancer cachexia-associated increases in catabolic CL.

We have previously identified that FcγR expression is altered in murine models of cachexia and in patient PBMCs (55). To test FcγRs’ contribution to increased ICI CL in the LLC model, we utilized hIgG1 with a D265A point mutation, which abrogates all binding capabilities to FcγRs. Our PK study utilizing D265A and control hIgG1 in TF and LLC TB mice showed no differences in CL rates between TF mice receiving hIgG1 and D265A. However, comparisons between LLC TB groups receiving hIgG1 or D265A showed a statistically significant decrease in CL when FcγR interaction is removed with the D265A mutation, suggesting that FcγR interaction contributes to cancer cachexia-associated increases in catabolic CL. In other words, in healthy conditions, FcγRs are not a significant pathway of IgG catabolic CL, but they significantly contribute to enhanced catabolic CL in the context of LLC tumor-induced cachexia. Additionally, the antibodies used in this PK study have no target binding capabilities in mice and were given as a single dose, suggesting that the changes observed in antibody PK cannot be attributed to target-mediated drug disposition nor anti-drug antibody formation, and rather the differences are primarily driven by other, non-specific, catabolic pathways.

The liver has been shown to be a major site of antibody catabolism (56). Further, Mangeat et al. observed that radiolabeled hIgG1 had 4 fold higher liver deposition and accumulation compared to the FcγR null binding mutant when given intravenously, suggesting the importance of liver FcγR on IgG disposition and PK (57). Due to the liver’s suggested role in antibody PK, there was a need to further investigate mechanisms of FcγR-mediated catabolism in this tissue. The major FcγR expressed in the liver is RIIb (63). It is estimated that in mice, 75% of total body RIIb is expressed in the liver, with LSECs accounting for 90% of liver RIIb expression, or ~67.5% of total body RIIb expression (49). It is also understood that therapeutic antibody interaction with LSEC RIIb can impact antibody PK, CL, and efficacy (68, 69). Our immunofluorescence data demonstrates near total colocalization between infused hIgG1 and RIIb in the hepatic sinusoids, suggesting an association of infused hIgG1 with LSECs. It was then hypothesized that LSEC RIIb expression was altered in association with cancer cachexia in our pre-clinical tumor models. Our immunoblot data shows a trend of increased liver RIIb expression in the LLC model, but not in the TB, non-cachectic, MC38 model that displays no changes in ICI CL (39). Given these observations we sought to study the contribution of RIIb alone towards cachexia associated antibody CL increases by whole body RIIb knockout.

It was hypothesized that the magnitude of change in hIgG1 CL between TF and LLC TB groups would be reduced in RIIb -/- mice compared to WT mice, due to the lack of RIIb mediated CL pathways in knockout mice. However, when comparing the CL of WT and RIIb -/- mice, there was no significant impact of RIIb knockout on the CL of hIgG1. This data would not support the hypothesis that RIIb is a significant contributor to cachexia-associated antibody CL increases. Rather, it suggests IgG-FcγR interactions beyond RIIb, or specifically the activating FcγRI, FcγRIII, and/or FcγRIV interactions are important for antibody CL in the context of cachexia. Future investigations will explore potential contribution from Kupffer cells in the liver, as they also have considerable FcR expression, and other tissues involved in antibody CL, such as the spleen, as it is possible the liver may not be the main site of increased antibody CL observed in the LLC model. Future studies will also include FcγRI, FcγRIII, and FcγRIV specific knockouts to better understand the importance of each activating FcγR in cachexia associated increases in antibody CL. In addition, the increased ICI CL observed in LLC TB mice may result from the combined contribution of FcγR and FcRn, which is abundantly expressed in the liver (69). Previous investigations observed increased FcRn expression in cancer cachexia, which was contrary to hypotheses, and potential interactions of FcγR and FcRn have not been studied previously in the context of cachexia (55, 70).

Importantly, our study utilized a pan RIIb -/- model, where loss of RIIb in myeloid cells may lead to compensation by other receptors including upregulation of Fc effector functions mediated by activating FcRs (43). It has also been reported that lack of functional RIIb on B cells leads to increased IgG production and circulating levels of IgG in RIIb -/- mice (71), which could potentially affect FcRn-mediated recycling of IgG1. Thus, future investigations will utilize endothelial cell-specific and myeloid cell-specific RIIb knockout mice to better understand the contribution of RIIb towards antibody PK from each respective cell type. Differences were also observed in skeletal muscle mass between WT and RIIb -/- mice even when normalizing muscle mass to initial body weight (day -1) for male gastrocnemius (p=0.001), male quadricep (p=0.001), and female gastrocnemius (p=0.003). While the magnitude of tumor induced changes in skeletal muscle mass was not affected by genotype, it is important to note the differences in muscle mass between genotypes when interpreting these results. Previous investigations have reported the expression of FcγRIIb on skeletal muscle microvascular endothelium and the role of FcγRIIb in skeletal muscle glucose homeostasis (72). Genetic knockout of FcγRIIb on skeletal muscle microvascular endothelium impacting skeletal muscle growth may offer one possible explanation to the observed effect of genotype on skeletal muscle mass in mice. These differences in muscle and tissue mass as a result of genotype were not anticipated, and future studies should explore mechanisms of FcγRIIb impact on skeletal muscle growth and regulation. However, since RIIb -/- genotype had a negligible effect on antibody PK, it is unlikely that differences in starting skeletal muscle mass between genotypes had a significant impact on the study interpretation.

Given the importance of antigen binding and subsequent immune complex formation to RIIb-mediated uptake in LSECs (49, 68), future investigations will include anti-murine PD-1 antibody as antigen-antibody complex formation could potentially impact magnitude of FcγR effector functions. Future studies will be completed with a focus on further understanding the involvement of RIIb upon antibody PK and catabolism.

Literature has shown that in preclinical models, modulating anti PD-1 Fc affinity for FcγR drastically altered anti-tumor outcomes, with optimal anti-tumor efficacy observed in FcγR null binding variants (52, 53). The development of next generation ICIs, such as penpulimab, tislelizumab, prolgolimab, and others, that possess structural mutations that either greatly reduce or abrogate binding to FcγRs, operate on these findings to maximize efficacy and reduce off target Fc effector function complications (48, 73–76). Tislelizumab is an anti PD-1 antibody with a structural mutation to abrogate FcγR binding (Fc silencing) and is currently approved by the FDA as a monotherapy treatment for inoperable or metastatic esophageal squamous cell carcinoma (77, 78).

While it is well accepted that IgG4 based anti-PD1 targeted therapeutics such as pembrolizumab and nivolumab that retain FcγR interaction display changes in CL over time (time varying clearance) hypothesized to be related to disease status (25), this phenomenon was reported to be negligible in tislelizumab (79). Differences in observed time varying CL between tislelizumab and nivolumab/pembrolizumab are important when interpreting the results of this study, as it could suggest that disease-mediated contributions to ICI CL are mitigated when removing FcγR affinity in tislelizumab. Studies directly comparing the pharmacokinetics and efficacy of pembrolizumab or nivolumab versus anti PD-1 with Fc silencing mutations in humans would be needed to further support this. As it stands, no such direct comparison exists, thus limiting the field’s understanding of FcγR contributions to clinical ICI efficacy and PK. Improved understanding of ICI FcγR interactions and their impact on clinical response could increase patient response rates by optimally tuning FcγR, and Fc effector functions.

Our findings suggest that under healthy conditions, FcγRs do not significantly contribute to antibody CL. However, cancer cachexia-associated signaling mediates changes in FcγR expression and/or function that result in a significant role for FcγR in catabolic CL of IgG antibodies. These results have the potential to explain disease-mediated changes in ICI CL in clinical populations and could suggest FcγR as a mechanistic link between ICI CL and response relationships independent of drug exposure, however further clinical study is required to support this.

Our studies include a number of limitations that should be taken into account. First, these experiments utilized hIgG1 antibodies while the majority of clinical anti PD-1 ICIs are built upon an IgG4 backbone (25). IgG4 antibodies are utilized as they are believed to have no affinity for FcγRs, but this idea has since been revised as human IgG4 retains affinity to human and murine FcγRs when measured by surface plasmon resonance (54). Additionally, studies have uncovered that the affinity of the Fc portion for FcγR does not necessarily correlate with the magnitude of Fc effector functions elicited (80). It is still possible that slight differences in affinity between IgG1 and IgG4 could complicate the extrapolation of this data to antibodies built upon IgG4, as a result these differences should be kept in mind when interpreting these data. However, engineering Fc mutations in future ICIs may circumvent these differences in affinity and their impact as next generation ICIs, as Fc silencing mutations are being evaluated in both IgG1 and IgG4 backbones (73).

It is also important to note that while removal of FcγR binding with the hIgG1 D265A mutation affected catabolic antibody in LLC TB mice, there was still a significant increase in CL between TF and LLC TB mice. This highlights that while FcγRs are a factor, there are still other non-FcγR-mediated processes occurring in the LLC model that drives the increased antibody catabolism. Furthermore, we did not evaluate CL of the D265A antibody in the RIIb -/- mice. It is therefore possible that the other non-FcγR-mediated processes somehow decreased CL of D265A compared to hIgG1. These non-FcγR-mediated processes in the LLC model will be a focus in future studies, as an understanding of these pathways of antibody catabolism are important to the field’s knowledge of antibody pharmacology and may have clinical importance.

Lastly, while our studies were limited to elevated catabolism in the context of cancer, and specifically, cancer cachexia, models of other diseases associated with cachexia, such as chronic kidney disease (81), sepsis (82), and others (83) are required to confirm that disease-mediated changes in FcγR contribute more universally to heightened IgG catabolism. Understanding FcγR interactions are vital for the development of all antibody therapeutics but understanding disease-associated FcγR expressional changes and their associated impact upon immunotherapy may have far reaching implications to other diseases and therapeutic modalities.

## 5 Conclusion

Tumor induced inflammation and cachexia in the LLC model alters FcγR receptor expression (55). The data reported here suggests that under physiological conditions, FcγRs are not a significant contributor to antibody catabolism. However, in LLC TB mice there is an increase in the CL of antibodies that can be partially explained by antibody Fc domain interaction with FcγRs, as the magnitude of antibody CL increase between TF and LLC TB mice was reduced with an antibody that cannot bind to FcγR. This observation could not be explained by RIIb alone, as there was no observed difference in antibody CL in RIIb -/- mice but instead suggests the importance of composite FcγR and/or FcRn contribution to the disease-associated CL increases. Abrogation of all FcγR binding shows increased anti-tumor efficacy in pre-clinical cancer models, and the importance of these findings are being illustrated with the arrival of new anti PD-1 therapies receiving FDA approval that contain FcγR null binding modifications (52, 77, 84, 85). This study highlights FcγR as a pathway of disease-mediated increases in catabolic CL of IgG antibodies and highlights the need to further elucidate mechanisms of altered FcγR expression/function, as well as an understanding of aberrant FcγR expression/function’s ability to predict patient ICI CL and efficacy.

## Data Availability

The raw data supporting the conclusions of this article will be made available by the authors, without undue reservation.
