# Supplementary figures and images for: Fc gamma receptor binding modulates IgG clearance in cancer cachexia
Source: Front Immunol. 2026 Mar 31;17:1676732. doi: 10.3389/fimmu.2026.1676732 (PMC13076527; doi:10.3389/fimmu.2026.1676732)

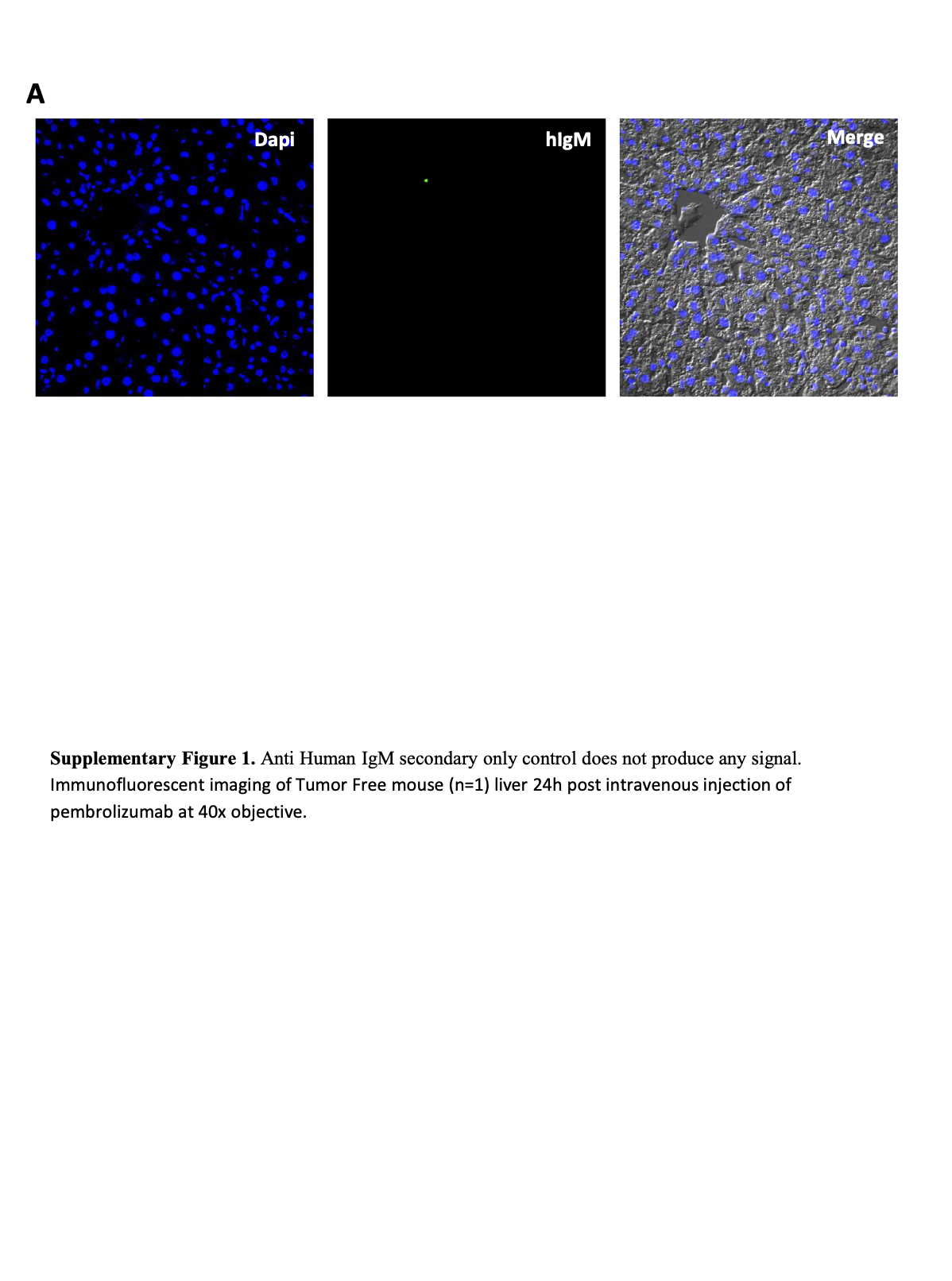

Supplement: Supplementary file 1 [file Image1.tiff]

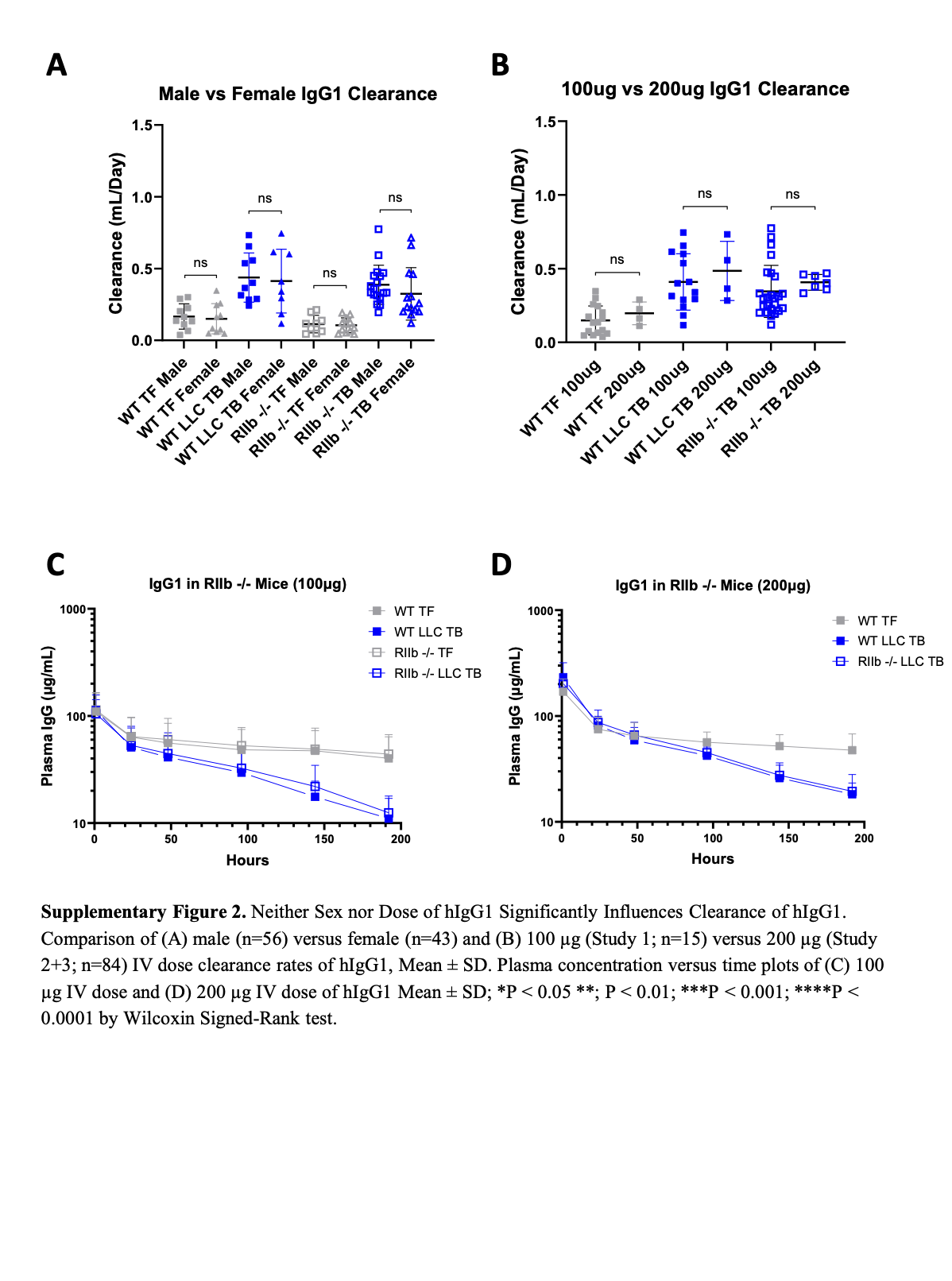

Supplement: Supplementary file 2 [file Image2.tiff]
